# A New Approach for Detection Improvement of the Creutzfeldt-Jakob Disorder through a Specific Surface Chemistry Applied onto Titration Well

**DOI:** 10.3390/bios2040433

**Published:** 2012-10-24

**Authors:** Caroline Mille, Dominique Debarnot, Willy Zorzi, Benaissa El Moualij, Isabelle Quadrio, Armand Perret-Liaudet, Arnaud Coudreuse, Gilbert Legeay, Fabienne Poncin-Epaillard

**Affiliations:** 1 LUNAM Université, UMR Université du Maine, CNRS n°6283, Institut des Molécules et Matériaux du Mans, Département Polymères, Colloïdes et Interfaces, av. O. Messiaen, 72085 Le Mans, France; E-Mails: caroline.mille.etu@univ-lemans.fr (C.M.); dominique.debarnot@univ-lemans.fr (D.D.); 2 Centre de Recherche sur les Protéines Prion, Institut de Pharmacie, B36, n°1 avenue de l’Hôpital, 4000 Liège, Belgium; E-Mails: willy.zorzi@ulg.ac.be (W.Z.); b.elmoualij@ulg.ac.be (B.E.M.); 3 Centre Mémoire de Ressources et Recherche, Laboratoire des Maladies à Prions, Groupement Hospitalier Est; Hôpitaux de Lyon 59 bd Pinel, 69677 Bron cedex, FranceCTTM, 20 rue Thalès de Milet 72000 Le Mans, France; E-Mails: isabelle.quadrio@chu-lyon.fr (I.Q.); armand.perret-liaudet@chu-lyon.fr (A.P.-L.); 4 CTTM, 20 rue Thalès de Milet 72000 Le Mans, France; E-Mails: acoudreuse@cttm-lemans.com (A.C.); gilbert.legeay@neuf.fr (G.L.)

**Keywords:** amphiphilic molecules, cold plasma, ELISA titration, prion protein

## Abstract

This work illustrates the enhancement of the sensitivity of the ELISA titration for recombinant human and native prion proteins, while reducing other non-specific adsorptions that could increase the background signal and lead to a low sensitivity and false positives. It is achieved thanks to the association of plasma chemistry and coating with different amphiphilic molecules bearing either ionic charges and/or long hydrocarbon chains. The treated support by 3-butenylamine hydrochloride improves the signal detection of recombinant protein, while surface modification with the 3,7-dimethylocta-2,6-dien-1-diamine (geranylamine) enhances the sensitivity of the native protein. Beside the surface chemistry effect, these different results are associated with protein conformation.

## 1. Introduction

The Creutzfeldt-Jakob disease was first described in 1920 by the German neurologists Hans Gerhard Creutzfeldt and Alfons Maria Jakob, who observed that some patients’ symptoms were similar to those of sheep scrapie. The brains of patients were riddled with holes like a sponge, and they named it spongiform encephalopathy. This disease subsequently took the name of the discoverers: Creutzfeldt-Jakob disease.

A healthy patient has non-pathogenic prion proteins, PrPc (Cellular Prion Protein), associated to the development of embryo nervous systems. Such proteins are involved in the adhesion and in the differentiation of cells, and act as antioxidant and apoptosis retarders. However, their most important role is to drive the folding of proteins, allowing the latter to be or not to be functional. This peculiarity has led to the development of Creutzfeldt-Jakob disease [1]. In addition, prion diseases are genetically transmitted in 10% of individuals to another [2]. This disease is characterized by dementia associated with motor abnormalities resulting from the presence of infectious prion protein PrPsc (Prion Protein Scrapie) [3]. This protein is an unconventional pathogen because it is devoid of nucleic acid, which holds the normally infectious information [2,4,5]. The infectious prion protein (molecular weight = 30 kDa) is involved in all diseases of spongiform encephalopathies, human or animal. It is an isoform resulting from the modification of the prion protein PrPc [6]. PrPc has a structure with 43% α helix and 3% β sheets, while PrPsc has 30% α helix and 43% β sheets [7,8]. The PrPsc could modify its own pathogenicity, as with any self-chaperone molecule that is able to spontaneously change its own conformation [9,10]. A glycannic co-factor could also help to spread the transmission of the infectious agent [11]. The PrPsc is not sensitive to proteolysis by protease K nor to different techniques of disinfection and sterilization (thermal, chemical, *etc*.), allowing its degradation into several sequences and digestion by the cell. This could be due to the larger number of β sheets that stabilize the protein and confer resistance to enzymes. The infectious prion protein exponentially multiplies itself within neurons, mutating through a system of unfolding/refolding healthy prion proteins into contact [12,13]. 

Definitive diagnosis of Creutzfeldt-Jakob disorder (CJD) is rather difficult in living patients. Unfortunately, clinical signs depend on the etiologic form of CJD (sporadic, hGH and v-CJD). The post-mortem examination of the brain is needed to formally give the diagnosis of CJD with characteristic neuropathological lesions, immunodeposits of PrPsc and immunodetection of PrPres. Assays (ELISA or western-blot) for PrPres detection are currently dedicated to cerebral or peripheral tissues, such as tonsils, spleen or intestinal nodes in v-CJD. It has become clear that more sensitive schemes would be needed in order to detect PrPres in cerebrospinal fluid (CSF) or blood, even in the clinical phase of the disease. At this stage of knowledge, it appears that a blood-screening test for v-CJD may be imminent [14]. Since blood transfusion is now highly suspected to carry a risk for v-CJD, the priority is to the preclinical blood detection of PrPsc/PrPres. New tools in the way of concentration of PrPsc are needed; for example, aptamers alone or in combination with other ligands would be of great interest [15,16]. Previous studies described the advances in screening test development for prion diseases, among which there are the fluorescent correlation spectroscopy [17], Seprion ligand [18], conformation-dependent immunoassay [19], time resolved fluorescence spectroscopy [20] and PMCA [21]. It has also been mentioned that immuno-PCR is a unique method capable of achieving a low level of detection [22]. In a previous study, we showed that bovine PrPres can be detected with high sensitivity by immuno-quantitative polymerase chain reaction, also called iqPCR [23]. This technology, previously described by Zorzi *et al*. in the patent EP1232283 [24], couples an antibody detection step similar to an ELISA with nucleic acid amplification by real-time PCR procedure. The detection threshold of iqPCR is lower than classical ELISA for recombinant and infectious bovine prion protein [23]. In another work, we assessed the sensitivity and specificity of iqPCR for the detection of PrPres in the brain of CJD patients. We have compared samples from the middle frontal gyrus of seven patients with sporadic CJD and seven controls using iqPCR, immuno-histochemistry (IHC), ELISA and Western Blot. The iqPCR was specific in all cases and appeared at least 10-times more sensitive than the other standard methods. Therefore, we proposed iqPCR as one of the choice methods for PrPres detection in brain surgical biopsy and autopsy specimens [25]. The high sensitivity of iqPCR makes it a powerful method for the detection of small deposits of PrPres in the central nervous system and could be useful for the work-up of cases of spongiform encephalopathy with minimal amounts of PrPres, for which IHC, Western Blot and ELISA fail to provide a definite diagnosis. It could be particularly useful on brain biopsies performed in the context of subacute dementia, as such biopsies typically sample areas such as the anterior frontal cortex, where CJD may manifest with only very low deposits of PrPres. Another way is proteinopathy to better identify the chemical and biochemical behavior of proteins, and this could be a sensitive method of diagnosis [8]. The major problems of safety and public health make these techniques for detecting neurodegenerative disease a huge economic issue, not only in order to understand the clinical signs, but also to improve the sensitivity of the detection system, and then to allow ante-mortemdetection [26]. Our goal is to show that better titration could be achieved by controlling the non-specific adsorption of proteins and therefore, also controlling the surface chemistry of the inner surface of ELISA wells used for the detection and dosage of prion proteins.

The interactions between substrate and biomolecule are mostly controlled by the mechanical anchoring (roughness of the surface), by chemical, electrostatic, van der Waals, and hydrogen bond interactions (hydrophilic-hydrophobic balance and charge effect) that may enhance the bioadhesion and therefore, the detection threshold of ELISA titration [27]. For such a purpose, functionalization of the inner surface of titration well based on polypropylene (PP) was studied, thanks to different processes using environment-friendly wet chemistry of amphiphilic molecules and plasma chemistry [28]. In this study, plasma chemistry acts as an activation step allowing the reactive species formation onto the PP surface, then the grafting of amphiphilic molecules after dipping the plasma-modified surface. The hydrophilic charge character and, partially, the topography are induced by such molecules. Because of their specific auto-organization behavior, the coated molecule should have a certain orientation, with the hydrophilic head attracted by the hydrophilic plasma-treated surface. A multi-layer deposition is expected with sequences in head-tail-tail-head association leading to a hydrophilic extreme surface of the coated substrate.

## 2. Experimental Section

### 2.1. Materials

The polypropylene supports (PP, 7 cm^2^ plates and strips of eight wells) of polypropylene (Θ_H2O_ = 99°, γ^t^ = 30.8 mJ·m^–2^, γ^nd^ = 0.6 mJ·m^–2^) were manufactured by the company EUDICA (Annecy, France). 

The hexatrimethylammonium bromide (T1), 3-buten-1-amine hydrochloride (T2) and (E)-3,7-dimethylocta-2,6-dien-1-amine (geranylamine) (T3) were commercial products (Aldrich) used without other purification. 


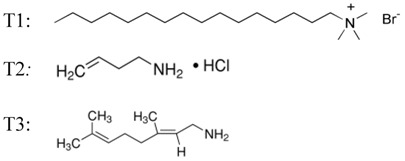


### 2.2. Cold Plasma Treatment

The plasma treatment fully described in [28,29] was run in a radiofrequency (RF) plasma reactor designed in the PCI laboratory. Experiments were performed in a RF plasma reactor. The discharge chamber was made of aluminum and had a volume of approximately 9 L. Commercially available, highly purified He (<5 ppm of O and <1 ppm of H_2_O) was leaked into the discharge chamber through a precise flow controller at variable flows. The powered electrode was connected to a matching network that was in turn connected to a 13.56 MHz RF generator. Samples were mounted on the bottom of the discharge chamber.

### 2.3. Amphiphilic Molecular Coating

The products were dissolved in distilled water under ultrasonic conditions for 20 min at 37 °C, except for T3, where 10 volume% of ethanol were added. Following exposure to plasma, the sample was immersed 5 h in an aqueous solution of T2 (1 mM) and T3 (1 mM) for 12 h T1 (1 mM).

After a certain duration, the samples were removed and dried 5 h under a laminar flow hood, then packaged under ambient atmosphere in a sterile polyethylene bag.

### 2.4. Surface Characterization

In order to evaluate the wettability of surfaces before and after functionalization, the measurements of water and diiodomethane contact angles were carried out using a goniometer from Rame HART Inc. (model: 100-00-230), then the non-dispersive (γ^nd^), total (γ^t^) energies of each surface were calculated using the Fowkes method—Dupre-Young on the average values of contact angles [30,31]. For each tested substrate, the values of the contact angles observed is the average of six measurements made using three drops of each liquid, 3 µL of ultrapure water and 1.5 µL of diiodomethane.

Compared to the virgin PP, the three modified surfaces were more hydrophilic, due to the attachment of oxygen and nitrogen atoms, PP-T1 having the highest value of non-dispersive energy because it was also bearing bromide atoms. The roughness of PP-T1 and PP-T2 was higher than that of the virgin PP, but still low compared to the polymeric surfaces. Also note that without any plasma treatment, the amphiphilic coating did not adhere onto PP substrate. Surface chemistry of new surfaces described through XPS analysis was given in [27,28,29].

### 2.5. ELISA Titration

#### 2.5.1. Recombinant Human Prion Protein (PrPrechum) Sandwich ELISA Protocol

Plastic well (Roboscreen, Leipzig, Germany) surfaces were precoated with 10 µg∙mL^–1^ of capture antibody Saf32 (a gift from Jacques Grassi, CEA, Paris, France) diluted in carbonate buffer (pH 9.4) at 4 °C overnight. The next day, the wells were emptied, washed three times (washing buffer: 50 mM Tris, 150 mM NaCl, 0.5 mM Tween 20 0.1%), blocked for 1 h with 200 µL of blocking buffer (washing buffer containing 10 g∙L^–1^ bovine albumin), and rinsed again. The human recombinant prion protein (Roboscreen, Leipzig, Germany) was incubated in the wells at concentrations from 0 ng∙mL^–1^ to 50 ng∙mL^–1^ for 1 h at 37 °C. The strips were washed with PBS and then incubated with the biotinylated detection antibody 4F7 (1 µg∙mL^–1^) for 1 h at room temperature. The wells were again washed three times with PBS. The biotinylated detection antibody coupled 4F7-biot (Roboscreen, Leipzig, Germany) diluted in PBS (pH 7.4) (1 µg∙mL^–1^) was coated for 1 h at 37 °C. Then, the wells were washed three times, blocked for 1 h with 200 µL of blocking buffer and rinsed again. Peroxidase-conjugated streptavidin (Dako, diluted 1:7,500) was added to each well and incubated for 30 min at room temperature. After five washes in PBS, the residual peroxidase activity was measured by means of chromogenic reaction with a solution containing equal amounts, by volume, of 3,3′,5,5′-tetramethylbenzidine and H_2_O_2_ (BD PharMingen). After incubation for 30 min in a dark at room temperature, the reaction was stopped by addition of 1 M H_2_SO_4_. The absorbance of the reaction mixture was measured at 450 nm with an automatic reader instrument (BioTek ELX800NB). The limit of the detection is calculated by measuring the optical densities of the negative control (background signal) and by multiplying this value by a factor of about two to three. This value of about two to three indicates the limit of detection, or the cut-off of the ELISA experiments aimed to determine the specificity and sensitivity of the diagnostics test.

#### 2.5.2. PrP-DVE Sandwich ELISA Protocol

Human brain extract originated from Laboratoire des Maladies à Prions, Groupement Hospitalier Est, Hôpitaux de Lyon. Infectious prion protein was purified using the Purification Biorad Prion Kit, according to manufacturer’s instruction. Briefly, nervous tissue was homogenized for 45 s, and 200 µL of this homogenate was PK treated 10 min at 37 °C in buffer A. After stopping the reaction and adding 200 µL of buffer B, tubes were centrifuged for 5 min at 20,000 g. Immunodetection was operated on pellets previously resuspended in buffer C and heated 5 min at 100 °C in the same buffer. Plastic well surfaces were precoated with 10 µg∙mL^–1^ of capture antibody 3F3 (Roboscreen, Leipzig, Germany) dilute in PBS (pH 7.4) at 4 °C overnight. The next day, the wells were emptied, washed three times, blocked for 1 h with 200 µL of blocking buffer and rinsed again. The precoated, saturated strips were incubated with different dilutions (1:250, 1:500, dilutions) of human brain extract for 1 h at room temperature. The strips were washed with PBS and then incubated with the detection antibody 15F5-HRP (Roboscreen, Leipzig, Germany) diluted 1/10 in PBS (RoboScreen) for 1 h at room temperature. The wells were again washed three times with PBS. The 3,3′,5,5′-tetramethylbenzidine (75 μL), peroxide solution (45 μL) and 3 mL associated buffer (Roboscreen) were added to each well and incubated for 30 min at room temperature. After incubation, the reaction was stopped by addition of 1 M H_2_SO_4_. The absorbance of the reaction mixture was measured at 450 nm with an automatic reader instrument (BioTek ELX800NB).

## 3. Results and Discussion

In our previous study [32], we showed that a simple modification of the inner surface of the assay wells by plasma process led to an increase of the detection limit of the prion protein. So, we propose here to study the influence of more complex surface chemistry based on the coating by surfactant molecules on the same type of dosage.

### 3.1. ELISA Titration of Recombinant Human Prion Protein (PrPrechum)

In the first step, the efficiency of the surface modification of wells was evaluated through the detection of recombinant human prion protein (PrPrechum). This protein contains the main biological functions specific to the infectious prion protein without bearing the gene responsible for the disease.

**Figure 1:**
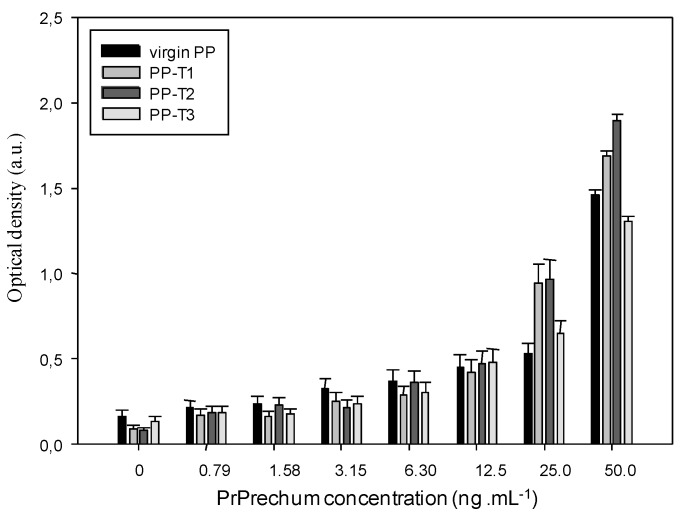
Dependence of the detection of PrPrechum protein on its concentration and the well inner-surface nature ((capture antibody) = 10 µg∙mL^–1^, (detection antibody) = 1 µg∙mL^–1^).

Figure 1 shows the evolution of the optical density obtained with virgin or coated (T1, T2 and T3) PP wells according to a range of increasing concentrations of recombinant prion protein in the presence of the Saf32 capture antibody (10 µg∙mL^–1^) and the 4F7biot biotinylated detection antibody (1 µg∙mL^–1^). 

The reported values are the average of five experiments performed at the same time to ensure reproducibility of results. These values are increasing with the antigen concentration. At PrPrechum concentration higher than 12.5 ng∙mL^–1^, the optical density with PP-T2, and with PP-T1 to a lesser degree, is higher compared to virgin PP. A surface bearing amphiphilic molecule with halogen counter ion seems to enhance the titration. Beside this observation, it should be noted that the background noise of the device—also due to non-specific detections corresponding to false positives, such as the affinity system primary antibody—secondary antibody at null antigen concentration—is lowered. Thus, without any prion molecules, the background noise (obtained without PrPrechum) with PP-T1 and PP-T2 are half that of the control (virgin PP), respectively, 0.090 a.u. and 0.079 a.u. against 0.165 a.u. 

**Figure 2:**
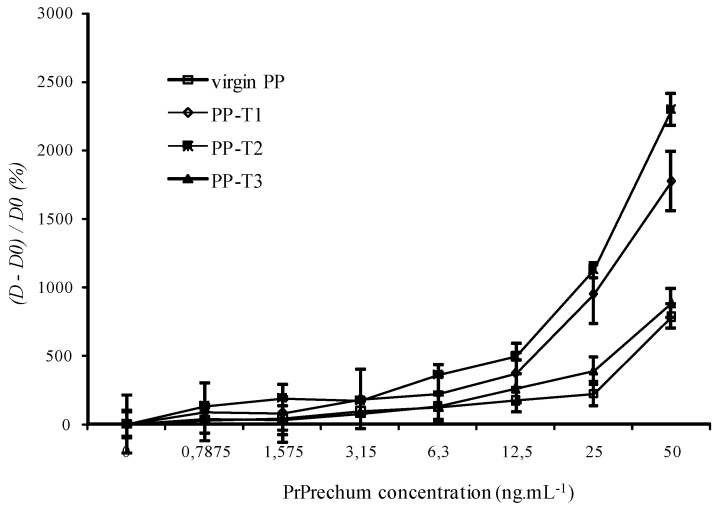
Dependence of the (D – D0)/D0 ratio on the well inner-surface nature and the antigen concentration. ((capture antibody) = 10 µg∙mL^–1^, (detection antibody) = 1 µg∙mL^–1^).

In Figure 2, the results are presented through a normalization step ((OD of measured sample-OD of control PP)/OD of control PP) corresponding to a sensitivity of the biosensor. Figure 2 confirms clearly that the treated substrates exhibit greater susceptibility to prion protein than the control, especially with T1 and T2 functionalization. The presence of a surface charge onto PP would therefore better fix the capture antibody by electrostatic effects and van der Waals interactions, and thus better detect the protein. From Figure 2, the detection threshold of PrPrechum is determined as 25 ng∙mL^–1^, 9.4 ng∙mL^–1^, 9.3 ng∙mL^–1^ and 14.2 ng∙mL^–1^ for virgin PP, PP-T1, PP-T2 and PP-T3, respectively. Indeed, the sensitivity is enhanced with T1 and T2 treatments, while T3 treatment has no significant improvement compared to virgin support, then it would confirm the need for a surface charge effect. The limit of detection (%∙ng^–1^∙mL^–1^) corresponding to the cross point between the slopes of insensitivity and sensitivity curves is also more important when T1 and T2 surface modifications are applied onto PP well: 47.9 for PP-T2 > 36.8 for PP-T1 > 22.5 for virgin PP > 19.8 for PP-T3. Compared to the results obtained with neuroproteins involved in Parkinson’s disease, it shows that the capture antibody has no affinity for the PP-T1 substrate. Thus, the sensitivity of the PP-T1 substrate in ELISA-assays is expected to be low. In contrast to that, the sensitivity of the PP-T1 substrate in the ELISA assays in the present work is as high as the one measured on the PP-T2 and PP-T3 substrates. This fact could be explained by different surface (physico-chemical or conformation) properties of both types of proteins in various biological media. 

### 3.2. Aging of New Biofunctional Wells

The aging of such modified surfaces must be controlled. Since in hospital surroundings the immunoenzymatic analyzes are not systematically carried out the same day as blood aliquot sampling, it is thus necessary to verify that the thin deposits have not aged and can be used for a longer time (for example, several months). 

**Figure 3:**
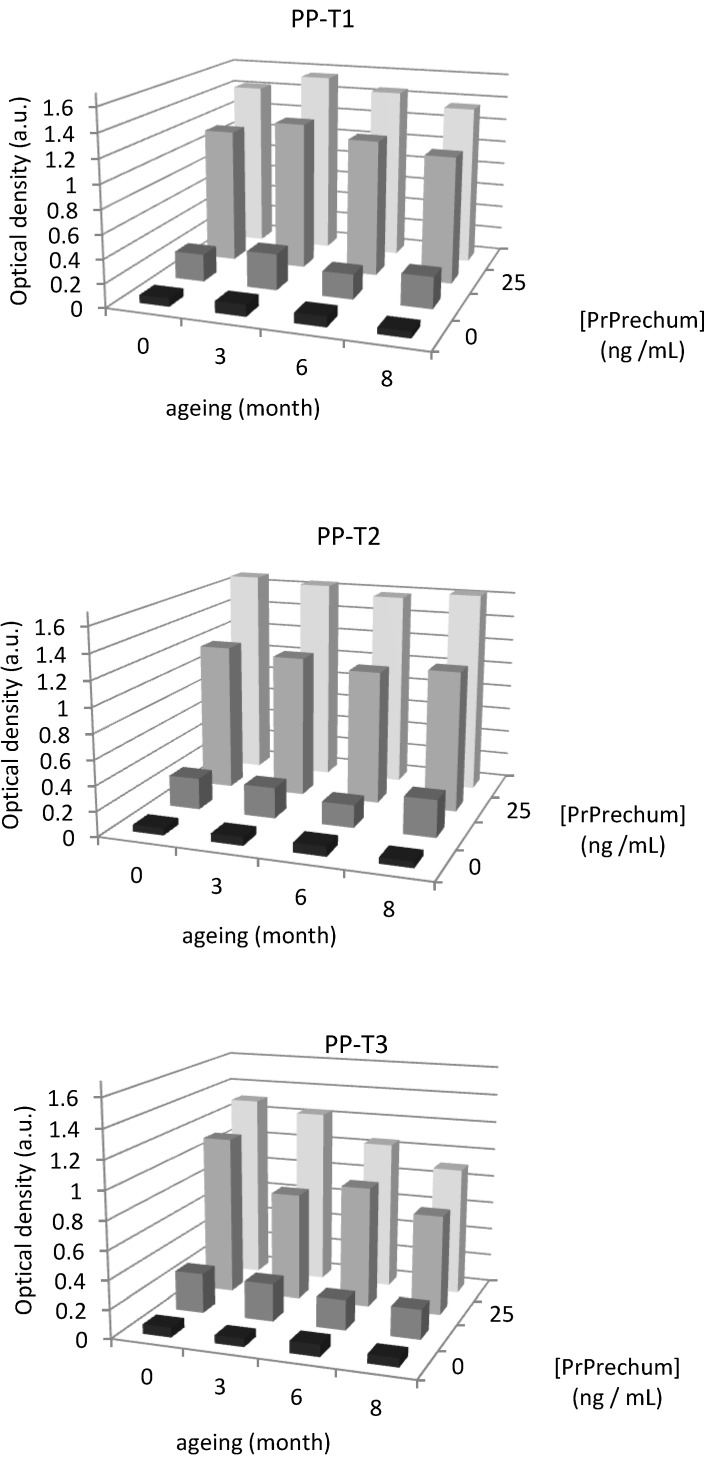
Evaluation of aging on the detection of PrPrechum protein with different surface chemistries of PP wells.

The physicochemical properties were previously verified, and it was shown that PP-T3 is the only surface that is aging. Accordingly, a series of eight samples for each treatment, T1, T2 and T3, was carried out followed by measurement of the contact angle each week during two months. The contact angles of PP-T1 and PP-T2 vary little over time. T1 treatment gives rise to an average value of the contact angle of 34° ± 1.8°, and T2 treatment a contact angle of 36° ± 0.8°. They, therefore, have both a very good stability over time. In contrast, the aging of treatment T3 has a drift over time, particularly after five weeks. The deposition of thin films seems to deteriorate over time. It is confirmed by the averaged values of contact angles that the error is significant: 49° ± 3.6°. In addition, aging of coatings were also evaluated in enzyme immunoassay detection in order to confirm the results obtained before. Figure 3 shows that the aging of materials *vis-à-vis* the detection of PrPrechum at various concentrations over a period of eight months, expressed as the optical density at zero, three, six and eight months of aging. The results show that the optical density analysis performed on the T3 support decreases over time, from 1.118 a.u. to 0.700 a.u. for the concentrations of 25 and 50 ng∙mL^–1^, respectively. Functional T1 and T2 show negligible loss of signal detection. These results are consistent with those obtained by contact angle measurements, showing an increase of hydrophobicity of surfaces treated with T3, while the other two treatments remain stable over time. This could explain the reduction in the detection of proteins. Since all biomolecules have a large number of possible conformations that occur depending on their environment, a more pronounced hydrophobic character would induce a new conformation and a lower affinity of the capture antibody towards the well surface, which results in a loss in detection.

### 3.3. ELISA Titration of Native Prion Protein (Pr-DVE)

To assess the efficiency of coated titration wells on the native protein titration, the same experiments as those performed on the recombinant protein were conducted on the proteins extracted from the CSF, even if the discrimination between the two forms based on proteinase K treatment is rather difficult. When the disease is symptomatically expressed, the CSF contains 10% of infectious prion protein and 90% of healthy prion proteins. It is then possible to collect samples from an external ventricular derivation placed on the patient.

**Figure 4:**
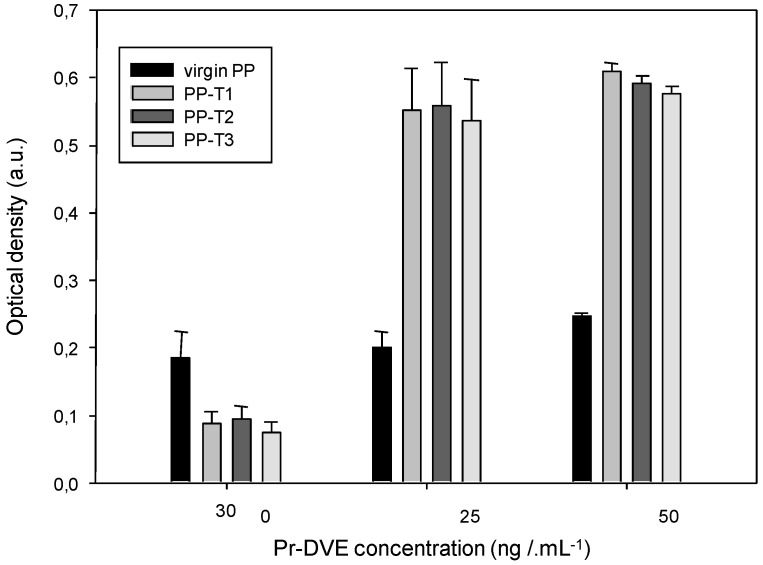
Dependence of the detection of Pr-DVE protein on its concentration and the well inner-surface nature ((capture antibody) = 10 µg∙mL^–1^, (detection antibody) = 1 µg∙mL^–1^).

The results of optical density values averaged over three experiments are shown in Figure 4. As observed before with PrPrechum, the optical density increases with the concentration of the native protein almost three-times compared to the titration run with virgin PP wells. The chemical modification of the inner-surface of the well also decreases the background noise, and therefore induces less non-specific adhesion of proteins. However, all the values of the optical density obtained with Pr-DVE are low compared with the ELISA titration of PrPrechum (Figure 1), probably due to the fact that the infectious prion protein represents only 10% of the global concentration of the native solution.

**Figure 5:**
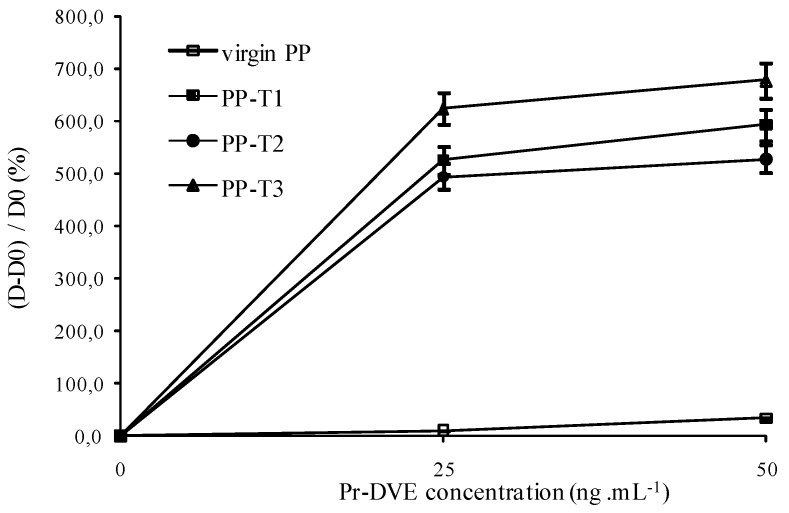
Dependence of the (D – D0)/D0 ratio on the well inner-surface nature and the antigen concentration ((capture antibody) = 10 µg∙mL^–1^, (detection antibody) = 1 µg∙mL^–1^).

The results of the (D – D0)/D0 ratio where D0 represents the obtained optical density without capture antibody for the different surfaces (virgin PP, T1, T2 or T3) (Figure 5) show a significantly improved sensitivity for the treated substrates compared to control and thus prove the effectiveness of such treatment. 

The saturation point is already reached at a concentration of 25 ng∙mL^–1^. Unlike experiments on the recombinant protein (Figure 2), the T3 treatment gives results slightly better than the other two treatments. These results illustrate the difficulty in considering the recombinant protein as a model of the infectious one. Differences between the properties of model and native proteins both in their conformations or hydrophilic character can be pointed out and lead to a singular affinity behavior. Even if the saturation takes place at 25 ng∙mL^–1^, the sensitivity (%∙ng^–1^∙mL^–1^) can be evaluated: 25.0 for PP-T3 > 21.1 for PP-T1 and > 19.8 for PP-T2 > 0.7 for virgin PP. Compared with PrPrechum ELISA, the sensitivities are almost divided by a factor of two, giving low values that may be associated to a dilution effect.

As for the PrPrechum protein, the aging of wells and the detection of Pr-DEV were studied over a period of eight months (Table 1). It appears here that the decrease of optical density is the most significant with the chemical functionalization T3, which can be explained by the aging of support as shown previously by angle measurements contact. 

**Table 1:** Aging of the ELISA well measured by Pr-DVE titration (optical density).

Aging duration (month)	(Pr-DVE) (ng·mL^–1^)	PP-T1	PP-T2	PP-T3
0	0	0.088 ± 0.018	0.094 ± 0.020	0.074 ± 0.016
	50	0.610 ± 0.026	0.591 ± 0.0114	0.576 ± 0.011
8	0	0.128 ± 0.027	0.103 ± 0.022	0.146 ± 0.031
	50	0.696 ± 0.013	0.512 ± 0.010	0.376 ± 0.007

### 3.4. Specificity of the ELISA Titration of PrPrechum and Pr-DVE

The sandwich ELISA detection system involves different biomolecules (antibodies, blocking agents, *etc.*) that all have a particular affinity for the molecule with which they must react during detection. When one of these biomolecules is missing, it is necessary to ensure that non-specific associations have not held and do not trigger a detection signal, leading mainly to false positives. Such an approach allows assurance that the treated supports do not enhance side-attachments or non-specific associations. During the detection protocol, the elimination of one of the main biomolecules (capture, detection antibodies, antigen protein) was used to assess the existence or otherwise of these non-specific adsorptions of molecules and the influence of well surface chemistry. Therefore, the ELISA titration of both proteins was run either in absence of capture antibody, antigen or detection antibody. 

**Figure 6:**
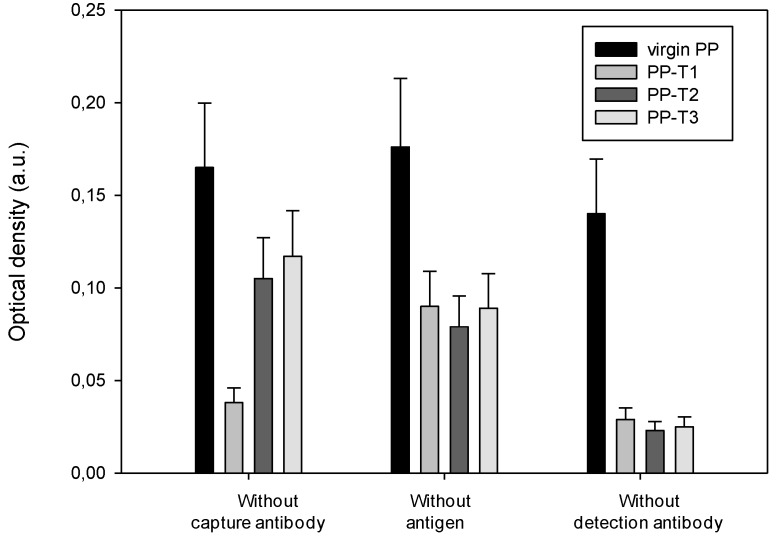
Evaluation of non-specific associations for each surface treatment during PrPrechum ELISA titration.

Figure 6 shows the values of optical density for PrPrechum titration recorded under different experimental conditions, where the antigen, capture and detection antibodies were each in turn removed from the protocol. 

First of all, the optical densities are relatively low—below 0.2 a.u.—whatever is the removed biomolecule. Moreover, the treated supports exhibit an optical density, lower than the virgin PP well. When the capture antibody is absent, PP-T1 has a lower optical density than the other two T2 and T3 treatments. PP-T2 and PP-T3 coated with the blocking agent seem to have a higher affinity towards the antigen than PP-T1 coating with the same blocking agent. The presence of positive charge attached by T1 repulses the antigen, thus limiting false positives. Moreover, since the optical density without antigen is in the same order whatever the surface chemistry of the well, the non-specific adsorption of the detection antibody is negligible, unlike with virgin PP well. The hydrophobic character of PP would promote the association of the two antibodies and induce detection. However, the presence of amines, free or charged, would reduce non-specific binding. In the last experiment, the detection antibody is not added to ensure that the complex streptavidin-HRP does not adsorb. The PP-T1, PP-T2 and PP-T3 present in that case detection signals below that one associated with virgin PP respectively 0.026 a.u. on average over the three supports against 0.14 a.u. Considering the values of detection signals obtained during the first experiment (without capture antibody), it seems worthwhile to retain the support treated with bromide hexatrimethylammonium (T1) in order to improve the detection of recombinant human prion protein.

**Figure 7:**
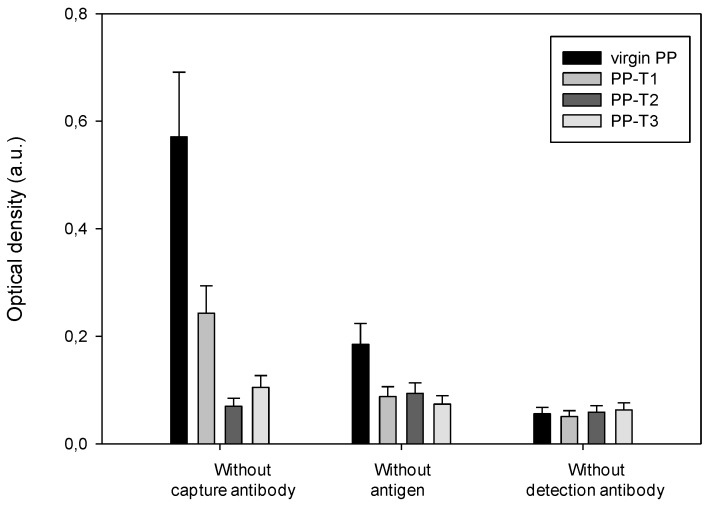
Evaluation of non-specific associations for each surface treatment during Pr-DVE ELISA titration.

In Figure 7, the variations of obtained optical density values based on the affinity of each biomolecule for each relative support treatment correspond to the Pr-DVE titration. The virgin PP well seems to fix the antigen and/or detection antibody in absence of the capture. Indeed, the value of the associated optical density is two to eight times greater than those of treated substrates. Compared to the other treatments, PP-T1 coated with bovine serum albumin (BSA) as a blocking agent gives rise to non-specific binding of the antigen and/or detection antibody. In absence of the antigen, the measured optical densities are low—below 0.1 a.u.—and of half value for the treated supports compared to the virgin well. This indicates a low detection antibody adsorption onto the capture antibody and/or the blocking agent. The presence of charges and amine groups on the surface of polypropylene therefore reduces background noise on the binding of secondary antibody to primary antibody. Moreover, the last experiment, corresponding to the absence of detection antibody, confirms that the weak obtained signal is due to the association of antibodies with each other. The T2 surface chemistry seems to be the most appropriate to the Pr-DVE detection because of the low optical densities in each experiment (below 0.1 a.u.), and therefore it decreases the background noise associated to non-specific binding.

## 4. Conclusions

The inner-surface of polypropylene wells used for ELISA titration was chemically modified and could either present hydrophilic or charged amino groups. These new titration wells were tested for the titration of native or recombinant prion proteins. The modified wells allow detection of the pathogen protein without false positives. Whatever is the chemical structure of the modified well, the ELISA sensitivity was emphasized compared to an ELISA titration run into a virgin PP well. However, depending on the extraction mode of the protein, the ELISA titration does not lead to the same result. In the case of titration of the recombinant protein, the highest sensitivity is achieved with PP-T2, while the native Pr-DVE protein presents a higher affinity towards PP-T3, a less ionized amphiphilic molecule with a longer hydrocarbon chain. The PP-T3 was shown to quickly age.
